# The Correlation and Risk Factors between Carotid Intima-Media Thickening and Alcoholic Liver Disease Coupled with *Helicobacter pylori* Infection

**DOI:** 10.1038/srep43059

**Published:** 2017-02-21

**Authors:** Qu Bao-Ge, Wang Hui, Jia Yi-Guo, Su Ji-Liang, Wang Zhong-Dong, Wang Ya-Fei, Han Xing-Hai, Liu Yuan-Xun, Pan Jin-Dun, Ren Guang-Ying

**Affiliations:** 1 Taishan Hospital, Taian, Shandong 271000, P. R. China; 2 Taishan Medical College, Taian, Shandong, 271000, P. R. China

## Abstract

The aim of this study was to explore the associations and differences in influencing factors between alcoholic liver disease (ALD) coupled with *Helicobacter pylori* infection and atherosclerosis and to determine whether there is a “double hit phenomenon” in atherosclerosis patients with ALD and *H. pylori* infections. Included cases (n = 160) were categorized into 4 groups: 41 cases of ALD coupled with *H. pylori* infections (group A), 35 cases of *H. pylori* infections without ALD (group B), 37 cases of ALD without *H. pylori* infections (group C), and 47 normal control cases (group D). CIMT was significantly greater in group A than in groups B and D (P = 0.005 and P = 0.001, respectively). The GLM univariate analysis found that CIMT was significantly greater in group A than in groups B, C and D (P = 0.018, P = 0.001 and P = 0.009, respectively). We found that BMI and ALT, AST and ApoB levels were independent predictors of CIMT (P = 0.000, P = 0.000, P = 0.012 and P = 0.014, respectively). ALD coupled with *H. pylori* infection may result in significant CIMT thickening, but *H. pylori* infection without ALD and ALD without *H. pylori* infection does not, suggesting that a “double hit phenomenon” occurs. Additionally, BMI, and ALT, AST and ApoB levels were independent risk factors for increased CIMT.

Four clinical characteristics that are generally more prevalent in patients with cardiovascular and cerebrovascular diseases are morbidity, disability, recurrence rate and mortality. These characteristics are also closely associated with atherosclerosis, which makes them an important subject of clinical research. Reports in the literature show that, regardless of the presence of fatty liver or *H. pylori* infection, increased carotid intima-media thickness (CIMT) alone can aggravate the progression of atherosclerosis to a certain extent. Previous studies have demonstrated that the prevalence of non-alcoholic fatty liver disease (NAFLD) is increasing worldwide as a result of rising rates of obesity^1^ and diabetes mellitus (DM)^1^,^2^. An increasing amount of evidence indicates that NAFLD is not only a potentially progressive liver disease but that it also has systemic consequences^3^. For example, it might increase the risk of atherosclerosis^4^ and cardiovascular disease^4^,^5^,^6^, and it may increase the risk of cardiovascular morbidity and mortality^7^. However, contradictory results have also shown that when NAFLD is not accompanied by insulin resistance, it is not associated with a carotid atherosclerotic burden^2^. Although the risk of ischemic stroke is higher in NAFLD patients, NAFLD itself is not independently associated with ischemic stroke^8^. Additionally, *H. pylori* infection has been suggested to be associated with atherosclerosis^9^, and it has also been implicated as a risk factor for atherosclerosis in association with stroke and other cardiovascular diseases (CVDs)^10^. Contradictory research findings have confirmed that *H. pylori* infection is not associated with CIMT^11^ or clinical coronary heart disease events^12^. Therefore, the effects of NAFLD and *H. pylori* infection on arteriosclerosis remain controversial. However, in clinical practice, we have found that many patients suffer from alcoholic liver disease (ALD) accompanied by *H. pylori* infection. However, whether their coexistence aggravates increases in CIMT is unknown. Limited data are available regarding the relationship between ALD and/or *H. pylori* infection and CIMT or the risk factors that might be associated with CIMT. Hence, we sought to explore the associations and differences in influencing factors between ALD coupled with *H. pylori* infection and atherosclerosis and to determine whether there is a “double hit phenomenon” in atherosclerosis patients with ALD accompanied by *H. pylori* infections. Furthermore, we provide suggestions” regarding methods to prevent and treat atherosclerosis.

## Materials and Methods

### Participants

From January 2012 to December 2015, participants were selected following an inquiry into their case history and history of drinking alcohol. The patients simultaneously received an ultrasonic abdominal examination and determine CIMT and a 13C-urea breath test [13C-UBT]) at Taishan Hospital in Shandong Province. Finally, we recruited 113 patients that met the criteria for chronic alcohol ingestion^13^. Among these participants, 78 met the criteria for ALD^13^, 76 met the criteria for *H. pylori* infection, and 47 met the criteria for having neither ALD nor *H. pylori* infections. A total of 160 cases were therefore included in the study and categorized into 4 groups: 41 cases of ALD coupled with *H. pylori* infections (group A), 35 cases of *H. pylori* infections without ALD (group B), 37 cases of ALD without *H. pylori* infections (group C), and 47 control cases (group D). A cross-sectional study was performed to explore the associations between ALD coupled with *H. pylori* infection and CIMT. Risk factors were evaluated by measuring BMI; blood lipids, including total cholesterol (TC), triglycerides (TGs), low density lipoprotein (LDL), and high density lipoprotein (HDL); blood apolipoproteins, including apolipoprotein A1 (ApoA1) and apolipoprotein B (ApoB); glucose; serum levels of hepatorenal function indicators, including alanine transaminase (ALT), aspartate aminotransferase (AST), gamma-glutamyltransferase (GGT), blood urea nitrogen (BUN), and creatinine (Cr); inflammatory cytokines, including C-reactive protein (CRP) and interleukin (IL)-6; and a lipid peroxidation product, malondialdehyde (MDA). *The* clinical protocol *conformed to the declaration of Helsinki*. Written informed consent was obtained from all patients prior to their participation. The protocol was approved by the clinical research ethics committee of Taishan Hospital in Shandong Province. The exclusion criteria for the subjects have been described in a previous publication^14^.

### Experimental apparatus and reagents

A color ultrasound system (GEV7 and LOG7; GE), an automatic biochemical analyzer (7080; Japan), an Enzyme Standard Instrument (Type ANTHOS 2010, Austria) and a 13 C infrared spectrometer (Type YH08, Anhui Yanghe Medical Instrument Equipment Co., Ltd.) were used for this study. The reagents used included a 13C-Urea Breath Test Kit (Shenzhen Zhonghe Headway Bio-Sci & Tech Co., Ltd.), a CRP kit (provided by the Beijing JiuQiang Company), an IL-6 kit and an MDA kit (provided by Shanghai Enzyme-Linked Immune Co. LTD; all kits were manufactured by R&D companies, USA).

### Ultrasonic investigation of liver and CIMT

Liver and CIMT analyses were performed by two ultrasonologists. After an overnight fast, an abdominal ultrasound examination was performed by an experienced ultrasonographer using a 3.5–5 MHz convex probe and a high-resolution B-mode ultrasound scanner. Then, the average CIMT was calculated from three separate values obtained from measurements of the vertical distance from the inner surface of the inner membrane to the external surface of the tunica media at 1 cm proximal to the common carotid artery bifurcation in the left and right common carotid arteries.

### Detection of *H. pylori* infection

On the same day as the general healthcare examination, a 13C-UBT test was performed after a fasting period of at least 6 hours and including 2 collection time points: baseline and 30 minutes after ingesting a 75 mg 13C-urea capsule diluted in 100 mL of boiled water. The infection status of the patient was defined by analyzing exhaled breath samples using 13C infrared spectrometry. A receiver-operating characteristic curve analysis was performed to define cutoff delta-over-baseline (DOB) values. DOB ≥4 was considered to indicate a positive reaction, and DOB <4 was considered to indicate a negative reaction.

### Laboratory tests

On the same day as the general healthcare examination, peripheral venous blood samples were collected after overnight fasting for at least 10 hours. Serum was collected via centrifugation at 3000 rpm for 10 min, and the samples were then stored at −70 °C. Serum levels of blood lipids, apolipoproteins, glucose, and hepatorenal function indicators were measured using an automatic biochemistry analyzer. Serum CRP levels were measured via immunoturbidimetry. Serum levels of IL-6 and MDA were measured using enzyme-linked immunosorbent assay (ELISA) kits. All tests were performed according to the manufacturers’ instructions.

### Statistical analysis

Categorical variables are expressed as the sample size (number of cases) and percentage (%), and all comparisons were performed using chi-square (x^2^) tests. Quantitative variables are expressed as the mean plus or minus the standard error of the mean (SEM) (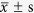). One-way ANOVAs were used to analyze multiple sample means. For multiple post hoc comparisons between groups, variables with normal distributions were analyzed using the LSD test, and variables without normal distributions were analyzed using Tamhane’s test. Then, using CIMT as a dependent variable and other parameters as covariates or independent variables, a general linear model (GLM) univariate analysis or a stepwise multivariate regression analysis were performed. All statistical analyses were performed using the SPSS statistical package (version 19.0 for Windows; SPSS Inc., Chicago, IL, USA). P values less than 0.05 were taken to indicate statistical significance.

## Results

Differences among the three or four groups in each experiment were analyzed to determine differences in sex, age, median alcohol drinking history, median daily alcohol consumption, BMI, CIMT, blood lipids, blood apolipoproteins, glucose, liver function indicators, renal function indicators and inflammatory cytokines. The results of these analyses are presented in Table 1. No differences were found in sex, age, median alcohol drinking history, or median daily alcohol consumption (all P > 0.05). However, significant differences were detected for BMI and CIMT among the four groups (F = 17.621, P = 0.000; and F = 4.435, P = 0.005, respectively). BMI values were significantly higher in groups A and C than in groups B and D (all P = 0.000), and CIMT was significantly greater in group A than in groups B and D (P = 0.005 and P = 0.001, respectively).

We found that differences in serum TC, TG, LDL and HDL levels among the groups were statistically significant (F = 7.115, P = 0.000; F = 11.362, P = 0.000; F = 3.579, P = 0.015, and F = 3.182, P = 0.026, respectively). Serum TC levels were significantly higher in groups A, B and C than in group D (P = 0.000, P = 0.024, and P = 0.019, respectively), serum TG levels were significantly higher in group A than in group B (P = 0.002), and serum TG levels were significantly higher in groups A and C than in group D (P = 0.000 and P = 0.007, respectively). Serum LDL levels were significantly higher in group B than in group D (P = 0.011), and serum HDL levels were significantly higher in group A than in group B (P = 0.016). No differences were found in ApoA1 and GLU levels (all P > 0.05). However, serum ApoB levels were found to be significantly different among the groups (F = 4.181, P = 0.007); they were significantly higher in groups A and B than in group D (*P = 0.001 and P = 0.028, respectively).

Median serum ALT, AST and GGT levels were also significantly different among groups A, B, and D (F = 3.574, P = 0.015; F = 3.202, P = 0.025; and F = 10.207, P = 0.000, respectively).

Serum ALT levels were significantly higher in groups A and B than in group D (P = 0.003 and P = 0.015, respectively), serum AST levels were significantly higher in group A than in group D (P = 0.022), Additionally, serum GGT levels were significantly higher in groups A, B and C than in group D (P = 0.007, P = 0.004 and P = 0.001, respectively).

Serum BUN levels were tested in each of the four groups (F = 2.924, P = 0.036); they were significantly higher in group A than in group B (P = 0.014) and significantly higher in group B than in group D (P = 0.015). However, there were no differences in serum Cr levels (P > 0.05) and no differences in CRP, IL-6 and MDA levels (all P > 0.05) among the four groups.

The GLM univariate analysis found significant associations between increased CIMT and BMI, ALT, AST and the four groups (F = 6.969, P = 0.010; F = 16.219, P = 0.00; F = 6.829, P = 0.011 and F = 4.507, P = 0.006, respectively); CIMT in group A was significantly higher than in groups B, C and D (P = 0.018, P = 0.001 and P = 0.009, respectively).

A stepwise multivariate regression analysis was performed to analyze the results using CIMT as a dependent variable and including all related independent variables, as shown in Table 2. We found that BMI, ALT, AST and ApoB were independent predictors of CIMT (P = 0.000, P = 0.000, P = 0.012 and P = 0.014, respectively).

## Discussion

Hepatosteatosis may be associated with coronary plaque instability and high fatty volume^6^. In the present study, we found that CIMT, which can be detected using ultrasound, is an early predictor of carotid atherosclerosis. Several previous studies have confirmed that CIMT is also associated with NAFLD^15^,^16^,^17^,^18^. In addition, the severity of NAFLD has been found to be significantly associated with CIMT^19^,^20^, and the presence of NAFLD should therefore be viewed as another probable independent factor that contributes to the development of carotid atherosclerosis^21^. However, contradictory findings in the literature suggest that fatty liver is not correlated with early carotid atherosclerosis in children^22^ and T2DM patients^23^. A limited amount of data is available in the literature demonstrating that patients with ALD display increased CIMT^18^,^24^. However, the results of the present study contradict this notion; we found that ALD was not associated with CIMT. A seven-year prospective cohort study revealed that microbial infections, such as *H. pylori* infection, can increase both CIMT and vulnerability to plaque development^25^. CIMT is higher in *H. pylori*-positive subjects than in *H. pylori*-negative subjects, and *H. pylori* infection might therefore play a role in atherosclerosis^9^,^26^, stroke^10^,^26^ and other CVDs^10^,^27^. CIMT in *H. pylori*-positive patients is greater than in *H. pylori*-negative subjects^28^, and in atherosclerotic stroke patients, infections with cytotoxin-associated gene-A-positive *H. pylori* strains have also been associated with greater CIMT^29^. Additionally, attempts at eradicating *H. pylori* infections might be reducing the emerging burden of CVD in Africa^27^, further supporting the notion that *H. pylori* may play a role in atherosclerotic processes. However, previous studies have come to opposite conclusions, indicating for example that *H. pylori*^30^ and the CagA strain^30^,^31^ were not major risk factors for early arteriosclerosis when the condition was assessed based on CIMT^30^,^31^. The data in the present study support the opposite viewpoint. Moreover, the results of a previous study^32^ demonstrate that NAFLD and metabolic syndrome have a synergistic impact on subclinical atherosclerosis, suggesting that individuals with both NAFLD and metabolic syndrome should be strongly advised to engage in CVD-preventing strategies. However, the relationship between AFLD in conjunction with *H. pylori* infection and CIMT remains unclear. In the present study, we showed that CIMT was significantly higher in patients with ALD coupled with *H. pylori* infections than in patients with *H. pylori* infections alone and normal controls. Additionally, the GLM univariate analysis found that there were significant associations between greater CIMT and BMI, ALT, AST and the four groups, and CIMT in patients with ALD coupled with *H. pylori* infections was significantly greater than in patients with *H. pylori* infections alone, patients with ALD without *H. pylori* infections and normal controls. Hence, the combination of ALD and *H. pylori* infection was associated with a significantly increased CIMT, suggesting that the coexistence of ALD and *H. pylori* infection results in a “double hit phenomenon”. In summary, AFLD patients with *H. pylori* infections have a higher incidence of increased CIMT, and measures aimed at preventing AFLD and eradicating *H. pylori* infections should therefore be recommended in these patients to prevent the development of early atherosclerosis.

The exact mechanisms underlying the increase in CIMT that result from the combination of AFLD and *H. pylori* infection remain controversial. Obesity and overweight are both components of metabolic syndrome, which is an important risk factor for atherosclerosis. An increased BMI^33^,^34^, visceral fat accumulation^22^ and childhood obesity^35^,^36^ are also significantly associated with greater CIMT. However, the relationship between BMI and ALD combined with *H. pylori* infection has not been determined. In the present study, we found that in patients with ALD or ALD coupled with *H. pylori* infections, BMI values were obviously higher than in the control group; but there was no clear difference in patients with *H. pylori* infections alone. These findings suggest that an increased BMI is one of the main contributors to ALD. Additionally, the regression analysis showed that BMI was positively related to CIMT, supporting the notion that BMI is a risk factor for higher CIMT and atherosclerosis.

Blood lipids and apolipoproteins are also important risk factors for atherosclerosis. A linear regression analysis of CIMT and TG and HDL cholesterol levels in a previous study indicated that these variables are associated with a greater risk of atherosclerosis and future adverse cardiovascular events^16^. Numerous studies have indicated that NAFLD^37^,^38^,^39^ and *H. pylori* infection^40^,^41^,^42^ may cause dyslipidemia by affecting TC, TG, LDL, HDL and OxLDL levels in addition to dysapolipoproteins, including ApoA1, ApoB and the ApoA1/ApoB ratio, and that these effects may impact the incidence and development of atherosclerosis. Our previous study confirmed that CIMT is associated with age and metabolic factors in patients with ALD^14^. However, the influence of ALD and *H. pylori* infection on blood lipid and apolipoprotein levels or their roles in increasing CIMT remain unclear. In the present study, we found that blood lipid and apolipoprotein levels, including serum TC, TG and ApoB levels, were significantly higher (and had the highest observed levels) in subjects with ALD coupled with *H. pylori* infections. In the other two groups, we observed different patterns. The regression analysis found that ApoB was positively correlated with CIMT, indicating that metabolic abnormalities resulted from the coexistence of ALD and *H. pylori* infection, which also contributed to increasing CIMT. ApoB was also an independent risk factor for increased CIMT. Hence, dyslipidemia and dysapolipoproteins might promote increased CIMT in patients with ALD coupled with *H. pylori* infection.

Currently, opinions on the associations between ALD, *H. pylori* infection and blood glucose levels are inconsistent. In the present study, we found that there were no differences in glucose levels in patients with ALD and/or *H. pylori* infections. The reasons for this finding remain unclear. Studies have shown that NAFLD is associated with impaired glucose regulation and T2DM^43^ and that glycated hemoglobin (HbA1C) is a determining factor for CIMT^22^. Additionally, CIMT-associated values, such as those used in cardiovascular risk assessments, were significantly higher in diabetic patients regardless of their degree of hepatosteatosis^44^. The prevalence of T2DM is higher in individuals with *H. pylori* infections^45^,^46^,^47^, and chronic *H. pylori* infection might induce an increase in HbA1c levels^45^ and a reduction in insulin secretion^45^. Additionally, eradicating *H. pylori* infection provides a benefit by improving insulin resistance in patients with normal blood glucose concentrations^48^. However, the opposite results have also been found in studies demonstrating that *H. pylori* infection does not affect fasting blood glucose and HbA1C levels but is associated with insulin resistance^49^. Another study demonstrated that being *H. pylori*- or CagA-positive does not affect whether a patient develops T2DM^50^. Therefore, the associations between ALD, *H. pylori* infection and blood glucose levels need to be examined in studies using larger sample sizes.

Inconsistent effects of *H. pylori* infection on liver and kidney functions have been reported in the literature. One study showed that liver enzymes decreased after patients received an *H. pylori* eradication regimen, suggesting that *H. pylori* infection plays a role in at least some patients with mild unexplained hypertransaminasemia^51^. In contrast, a patient’s *H. pylori* infection status was not found to be associated with either fatty liver disease or NAFLD, regardless of the patient’s sex, in another study^52^. Furthermore, the prevalence of *H. pylori* infection is higher in patients who have received long-term hemodialysis (>3 years)^53^, and in *H. pylori*-infected patients, IgG levels have been shown to be positively correlated with Cr clearance^53^,^54^. However, other studies support the opposing opinion that in patients with chronic renal insufficiency, *H. pylori* infection has nothing to do with the course of the disease or dialysis^55^ or with whether eradicating *H. pylori* has an effect on renal function^56^. The results of this study support this latter, opposing point of view. However, studies with larger sample sizes are needed to better explore this question in the future.

Recent studies have shown that ALT and inflammatory markers are associated with atherosclerosis and endothelial dysfunction. Serum ALT levels have been positively associated with the risk of carotid atherosclerosis in patients with NAFLD, suggesting that serum ALT levels could be used as a surrogate marker for cardiovascular risk in a specific clinical setting^57^. In patients with non-alcoholic steatohepatitis (NASH), serum GGT and ALT concentrations might be predictors of CIMT^58^. Increased serum ALT levels (even levels at the high end of normal) are associated with markers of CVD^59^, and they may therefore have prognostic value in assessing NAFLD patients, in whom they may potentially indicate an increased risk of CVD[62]. In patients with NAFLD, an elevated GGT concentration was associated with an increased risk of chronic kidney disease (CKD) among nondiabetic, non-hypertensive Korean men, regardless of the presence of metabolic syndrome^60^. Our results show that GGT serum levels were significantly higher in patients with ALD than in the control group, supporting this viewpoint. Additionally, serum ALT, AST and GGT levels were clearly higher in patients with ALD coupled with *H. pylori* infections than in patients with *H. pylori* infections alone or the control group, suggesting that ALD coupled with *H. pylori* infection may contribute to abnormal liver function, which itself can cause disorders in lipid metabolism that can initiate the progression of atherosclerosis. However, no previous studies have examined the associations between renal parameters and ALD coupled with *H. pylori* infection and CIMT. The existing literature shows that patients with NAFLD have higher BUN values^61^. In summary, a previous study showed that in NAFLD patients, an elevated GGT concentration was associated with an increase in the risk of CKD among nondiabetic, non-hypertensive Korean men, regardless of whether metabolic syndrome was present^62^. However, our study supports the notion that ALD and *H. pylori* infection specifically influence BUN levels.

Atherosclerosis is viewed as a chronic inflammatory disease. Many inflammatory cytokines play extremely important roles in processes involved in the formation and development of atherosclerosis. On the one hand, studies have shown that patients with NAFLD present higher serum levels of high-sensitivity CRP (hsCRP)^63^,^64^ and IL-6^62^,^64^. On the other hand, other studies have shown that the inflammation that follows *H. pylori* infection might promote the early stages of atherosclerosis in younger males^65^ and that infection with CagA-positive *H. pylori* strains may be a risk factor for the development of ischemic heart disease by contributing to a heightened inflammatory response or via other mechanisms^66^. *H. pylori* eradication has been shown to reduce the levels of pro-inflammatory cytokines, such as migration inhibitory factor and hsCRP^67^. *H. pylori* with CagA may induce significant increases in serum CRP and IL-6 levels^68^. In addition, *H. pylori* infection might increase the expression of IL-1β, IL-6 and TNF-α^69^. A study that produced contradictory results confirmed that serum IL-6 levels were not affected by *H. pylori* eradication in *H. pylori*-positive subjects^70^. The results of our study support the conclusion of Blum. However, our study also revealed that although serum CRP levels appeared to be different among the four groups, there was no significant difference between any two groups. In addition, a comparison of serum IL-6 levels among the four groups also showed that there was no clear significant difference. Based on these results, the association between AFLD with *H. pylori* infection and changes in the levels of inflammatory mediators remains controversial. Additionally, the results did not support the notion that inflammatory mediators are involved in increasing CIMT in patients with ALD accompanied by *H. pylori* infections. The exact relationship between these factors remains to be further explored.

MDA is the product of a lipid peroxidation reactions, induces cell toxicity and is a marker of oxidative stress. In patients with hepatosteatosis, MDA levels are significantly higher than in healthy subjects^71^. CIMT could be used as an indicator of the early atherosclerotic changes that are initiated by dyslipidemia and oxidative stress^72^. In duodenal ulcer patients with *H. pylori* infections, serum MDA levels have also been found to be significantly higher than in patients without *H. pylori* infections^73^. However, conclusions related to this association have been contradictory in that no significant correlation has been observed between oxidative stress markers and CIMT^9^. The results of the current study indicate that serum MDA levels were not significantly different among the four groups. Therefore, the role of oxidative stress in patients with ALD and *H. pylori* infections and the relationship between AFLD, *H. pylori* infection and CIMT remain to be confirmed by further research.

In conclusion, we show that while both ALD and *H. pylori* infection do indeed influence CIMT, the influence of these factors on CIMT was stronger when ALD was coupled with *H. pylori* infection. Hence, our results suggest that a “double hit phenomenon” exists between these factors. ALD and/or *H. pylori* infection may lead to an increase in BMI, abnormal blood lipid and apolipoprotein levels and abnormal liver and kidney function. Additionally, these changes are clearer in patients with ALD coupled with *H. pylori* infections. Furthermore, we found that BMI, ALT, AST and APOB are independent risk factors for increased CIMT. Hence, appropriate interventions aimed at controlling weight and ALT and ApoB levels should be helpful because they could simultaneously prevent increases in CIMT and the early stages of atherosclerosis. We suggest” that avoiding the development of ALD and eradicating *H. pylori* infections are important measures that be used to prevent and treat cardiovascular and cerebrovascular diseases.

Our study has certain limitations. First, the study sample size was small and biased because it contained a disproportionate number of male subjects. Second, confounding factors, such as drug use and other risk factors, were not considered.

## Additional Information

**How to cite this article**: Bao-ge, Q. *et al*. The Correlation and Risk Factors between Carotid Intima-Media Thickening and Alcoholic Liver Disease Coupled with *Helicobacter pylori* Infection. *Sci. Rep.* **7**, 43059; doi: 10.1038/srep43059 (2017).

**Publisher's note:** Springer Nature remains neutral with regard to jurisdictional claims in published maps and institutional affiliations.

## Figures and Tables

**Table 1:** Comparisons of sex, age, median alcohol drinking history, median daily alcohol consumption, CIMT, BMI, blood lipids, blood apolipoproteins, glucose, liver function, renal function and inflammatory cytokines among three or four groups (mean ± standard error).

Group	A	B	C	D	chi-square	or F P
N	41	35	37	47		
Sex_						
Male/Female					0.736	0.865
	36/5	32/3	34/3	41/6		
Age (y)					*0*.*021*	0.996
	46.37 ± 7.37	46.74 ± 6.69	46.72 ± 6.89	46.66 ± 6.75		
Mean alcohol drinking history (years)					1.059	0.351
	19.46 ± 7.06	16.67 ± 11.85	20.00 ± 7.66			
Mean daily alcohol consumption (g)					1.555	0.216
	86.00 ± 33.66	79.40 ± 23.11	85.75 ± 35.22			
CIMT (cm)					4.435	0.005
	0.084 ± 0.025^Δ#^	0.071 ± 0.019	0.076 ± 0.016	0.070 ± 0.016		
BMI (kg/m^2^)					17.621	<0.001
	26.70 ± 3.03^◇♦^	24.12 ± 2.26	27.20 ± 2.85^▲$^	23.81 ± 2.32		
Blood lipids (mmol/L)						
TC	5.62 ± 0.91^◇^	5.31 ± 0.64^Δ^	5.46 ± 0.97^♦^	4.83 ± 0.83		
					11.362	<0.001
TGs						
	1.94 ± 0.99^♦▲^	1.28 ± 0.51	1.60 ± 0.72^Δ^	1.12 ± 0.47		
LDL					3.579	0.015
	3.39 ± 0.86	3.46 ± 0.53^#^	3.42 ± 0.91	2.96 ± 0.88		
HDL					3.182	0.026
	1.36 ± 0.12^Δ^	1.26 ± 0.16	1.32 ± 0.08	1.36 ± 0.23		
Blood apolipoproteins (g/L) and glucose (mmol/L)						
ApoA1					0.243	0.866
	1.34 ± 0.24	1.38 ± 0.43	1.42 ± 0.31	1.36 ± 0.32		
ApoB					4.181	0.007
	1.11 ± 0.21*	1.06 ± 0.19^▲^	1.02 ± 0.26	0.94 ± 0.27		
GLU					1.215	0.306
	5.72 ± 0.91	5.61 ± 0.65	5.60 ± 1.09	5.41 ± 0.44		
Liver function (U/L)						
ALT					3.574	0.015
	37.63 ± 22.73^#^*	25.57 ± 11.36	32.37 ± 15.35	28.15 ± 17.93		
AST					3.202	0.025
	29.95 ± 14.32^&^	22.86 ± 4.88	26.66 ± 12.17	24.35 ± 9.70		
GGT					10.207	<0.001
	72.58 ± 62.22*^◇^	28.20 ± 14.84	71.94 ± 56.99^◇♦^	25.85 ± 12.43		
Renal function BUN (mmol/l)					2.924	0.036
	6.11 ± 1.30^#^	5.35 ± 0.79*	5.66 ± 1.45	5.97 ± 1.23		
Cr (μmol/L)					2.591	0.055
	65.16 ± 10.92	69.60 ± 12.13	63.54 ± 4.55	67.60 ± 10.27		
Inflammatory cytokines and lipid peroxidation product (ng/L)						
CRP					2.742	0.048
	5.71 ± 5.60	3.81 ± 1.96	4.05 ± 1.45	3.16 ± 1.62		
IL-6					0.418	0.741
	25.34 ± 16.09	22.64 ± 20.27	25.29 ± 21.29	27.74 ± 23.34		
MDA					2.163	0.095
	12.73 ± 9.77	8.87 ± 8.57	9.38 ± 7.42	12.91 ± 10.35		

BMI = body mass index, CIMT = carotid intima media thickness, TC = total cholesterol, TGs = triglycerides, LDL = low density lipoprotein, ApoA1 = apolipoprotein A1, ApoB = apolipoprotein B, GLU = glucose, ALT = alanine transaminase, AST = aspartate aminotransferase, GGT = gamma-glutamyltransferase, BUN = blood urea nitrogen, Cr = creatinine, CRP = C-reactive protein, IL-6 = interleukin-6, MDA = malondialdehyde.

CIMT: ^Δ^P = 0.005 (group A vs group B); ^#^P = 0.001 (group A vs group D).

BMI: ^◇^P < 0.001 (group A vs group B); ^♦^P < 0.001 (group A vs group D); ^▲^P < 0.001 (group C vs group B); ^$^P < 0.001 (group C vs group D).

TC: ^◇^P < 0.001, ^Δ^P = 0.024, ^♦^P = 0.019 (groups A, B and C vs group D).

TGs: ^♦^P = 0.002 (group A vs group B); ^▲^P < 0.001, ^Δ^P = 0.007 (groups A and C vs group D).

LDL: ^#^P = 0.011 (group B vs group D).

HDL: ^Δ^P = 0.016 (group A vs group B).

ApoB: *P = 0.001; ^▲^P = 0.028 (groups A and B vs group D).

ALT: ^#^P = 0.003; *P = 0.015 (groups A and B vs group D).

AST: ^&^P = 0.022 (group A vs group D).

GGT: *P = 0.007, ^◇^P = 0.004 (groups A and B vs group D); ^◇^P = 0.001, ^♦^P = 0.001. (group C vs groups B and D).

BUN: ^#^P = 0.014 (group A vs group B); *P = 0.015 (group B vs group D).

**Table 2:** Results of a stepwise multivariable regression analysis of CIMT as the dependent variable and related independent variables.

Independent variables	Nonstandardized coefficients		Standardized coefficients	*t*	*p*
	B	Std. Error			
Constant	0.004	0.014		0.287	0.775
BMI	0.002	0.001	0.317	3.925	<0.001
ALT	0.000	0.000	−0.441	−3.817	<0.001
AST	0.001	0.000	0.288	2.542	0.012
APOB	0.017	0.007	0.198	2.449	0.014
